# Supplementary material for: Content of Phenolic Compounds in Meadow Vegetation and Soil Depending on the Isolation Method
Source: Molecules. 2020 Nov 22;25(22):5462. doi: 10.3390/molecules25225462 (PMC7700638; doi:10.3390/molecules25225462)
Supplement: Supplementary file 1 [file molecules-25-05462-s001.pdf]

Table S1. List of phenolic compounds analysed.

Compound	Chemical formula	Symbol
Protocatechuic acid<br>(3,4-dihydroxybenzoic acid)<br>Supplier: Sigma-Aldrich	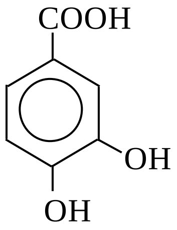	PA
p-Hydroxybenzoic acid<br>(4-hydroxybenzoic acid)<br>Supplier: Sigma-Aldrich	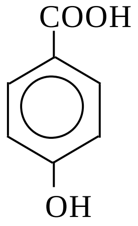	*p*-HA
Chlorogenic acid<br>3-(3,4-Dihydroxycinnamoyl)quinic acid<br>Supplier: Sigma-Aldrich	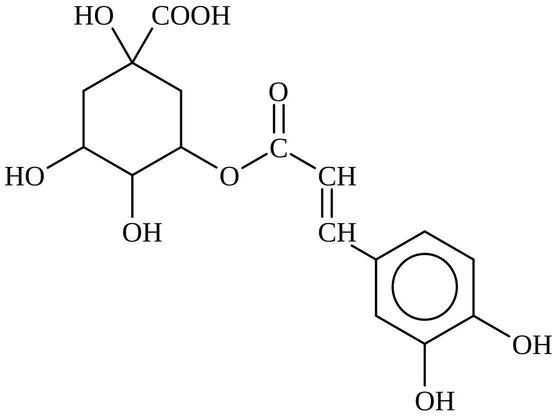	CHA
Vanillic acid<br>(4-hydroxy-3-methoxybenzoic acid)<br>Supplier: Fluka	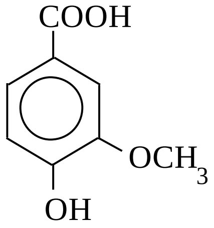	VA
Syringic acid<br>(4-hydroksy-3,5-dimethoxybenzoic acid)<br>Supplier: Fluka	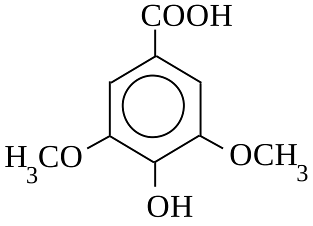	SYR

Caffeic acid<br>(3,4-dihydroxy-trans-cinnamic acid)<br>Supplier: Sigma-Aldrich	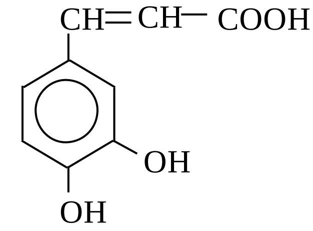	CA
Vanillin<br>(4-hydroxy-3-methoxybenzaldehyde)<br>Supplier: Sigma-Aldrich	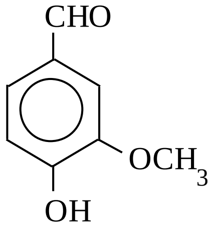	VAN
Syringaldehyde<br>(4-hydroxy-3,5-dimethoxybenzaldehyde)<br>Supplier: Fluka	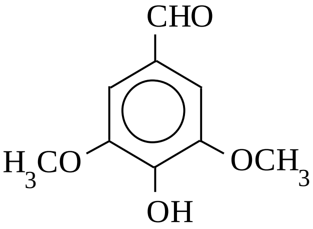	SYAL
p-Coumaric acid<br>(4-hydroxy-trans-cinnamic acid)<br>Supplier: Fluka	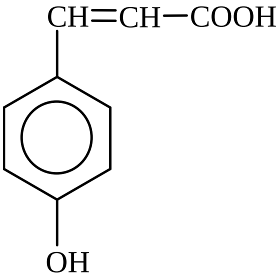	*p*-CO
Ferulic acid<br>(4-hydroxy-3-methoxy-trans-cinnamic acid)<br>Supplier: Sigma-Aldrich	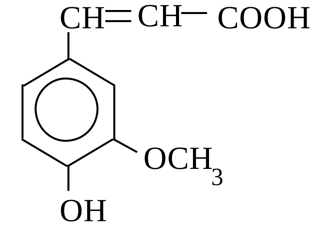	FEA
Salicylic acid<br>(2-hydroxybenzoic acid)<br>Supplier: Sigma-Aldrich	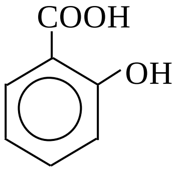	SA
